# Association between radiation pneumonitis and tumor response in patients with NSCLC treated with chemoradiation

**DOI:** 10.1186/1748-717X-9-217

**Published:** 2014-10-16

**Authors:** Michael P MacManus, David Ball, Rodney J Hicks

**Affiliations:** Departments of Radiation Oncology 1 and Molecular Imaging 2, Peter MacCallum Cancer Centre, St Andrew’s Place, East Melbourne, Vic 3002 Australia

**Keywords:** Radiation therapy, Lung cancer, Radiosensititivity, Tumour response, Positron emission tomograpy

## Abstract

Dang and colleagues recently reported in the journal that tumor response to definitive chemoradiation, as assessed using the RECIST criteria, and the risk of radiation pneumonitis were positively correlated in patients with non-small cell lung cancer (NSCLC). We had previously reported similar findings in a study that used positron tomography both to measure tumor response and to assess normal tissue toxicity in patients treated with chemoradiation for NSCLC. Taken together these reports suggest that radiosensitivity of normal tissues and tumors may be strongly linked in a proportion of patients with lung cancer.

## Correspondence

We read the recent article published in the journal by Dang and colleagues with interest [1]. These authors reported that tumor response to definitive chemoradiation, as assessed using the Response Evaluation Criteria In Solid Tumors (RECIST) criteria, and the risk of radiation pneumonitis were positively correlated in patients with non-small cell lung cancer (NSCLC). This is not the first time that such an association between tumor response and radiation-induced pulmonary toxicity has been reported in patients with NSCLC. In 2004, our group published the results of a study that investigated the relationship between positron emission tomography (PET)-detected inflammatory changes in irradiated normal tissues and clinical response at tumor sites in 73 patients treated with radical radiotherapy or chemoradiotherapy for NSCLC [2]. Radiation-induced inflammatory change was scored for normal tissues within the radiation treatment volume using a 0–3 grading scale. Metabolic tumor response was assessed using standardized visual metabolic response criteria. Increased fluorodeoxyglucose (FDG) uptake in normal tissues (radiotoxicity) was associated with a greater likelihood of complete or partial tumor response as assessed by both PET (p =0.0044) and computed tomography (p = 0.029). In a subsequent publication in 2011 we reported that PET-detected radiotoxicity and radiation pneumonitis were strongly associated [3]. FDG uptake in pulmonary tissues therefore appeared to reflect the inflammatory changes induced by radiation pneumonitis. Taken together, our results and the results reported by Dang and colleagues suggest that the intrinsic radiosensitivity of thoracic normal tissues and tumor responsiveness to chemoradiation may be related in at least a proportion of patients with NSCLC. These observations could potentially have useful implications for patient management. If the molecular basis of this phenomenon could be understood, it may be possible in future to estimate the likely radiosensitivity of both the tumor and the normal tissues of individual patients with NSCLC and use this information to better individualize therapy.

## Abbreviations

FDG: Fluorodeoxyglucose
NSCLC: Non-small cell lung cancer
PET: Positron emission tomography
RECIST: Response Evaluation Criteria In Solid Tumors.
